# Digitor/dASCIZ Has Multiple Roles in *Drosophila* Development

**DOI:** 10.1371/journal.pone.0166829

**Published:** 2016-11-18

**Authors:** Saheli Sengupta, Uttama Rath, Changfu Yao, Michael Zavortink, Chao Wang, Jack Girton, Kristen M. Johansen, Jørgen Johansen

**Affiliations:** Roy J. Carver Department of Biochemistry, Biophysics, and Molecular Biology, Iowa State University, Ames, Iowa, United States of America; CINVESTAV-IPN, MEXICO

## Abstract

In this study we provide evidence that the spindle matrix protein Skeletor in *Drosophila* interacts with the human ASCIZ (also known as ATMIN and ZNF822) ortholog, Digitor/dASCIZ. This interaction was first detected in a yeast two-hybrid screen and subsequently confirmed by pull-down assays. We also confirm a previously documented function of Digitor/dASCIZ as a regulator of Dynein light chain/Cut up expression. Using transgenic expression of a mCitrine-labeled *Digitor* construct, we show that Digitor/dASCIZ is a nuclear protein that is localized to interband and developmental puff chromosomal regions during interphase but redistributes to the spindle region during mitosis. Its mitotic localization and physical interaction with Skeletor suggest the possibility that Digitor/dASCIZ plays a direct role in mitotic progression as a member of the spindle matrix complex. Furthermore, we have characterized a P-element insertion that is likely to be a true null *Digitor/dASCIZ* allele resulting in complete pupal lethality when homozygous, indicating that *Digitor/dASCIZ* is an essential gene. Phenotypic analysis of the mutant provided evidence that Digitor/dASCIZ plays critical roles in regulation of metamorphosis and organogenesis as well as in the DNA damage response. In the *Digitor/dASCIZ* null mutant larvae there was greatly elevated levels of γH2Av, indicating accumulation of DNA double-strand breaks. Furthermore, reduced levels of Digitor/dASCIZ decreased the resistance to paraquat-induced oxidative stress resulting in increased mortality in a stress test paradigm. We show that an early developmental consequence of the absence of Digitor/dASCIZ is reduced third instar larval brain size although overall larval development appeared otherwise normal at this stage. While *Digitor/dASCIZ* mutant larvae initiate pupation, all mutant pupae failed to eclose and exhibited various defects in metamorphosis such as impaired differentiation, incomplete disc eversion, and faulty apoptosis. Altogether we provide evidence that Digitor/dASCIZ is a nuclear protein that performs multiple roles in *Drosophila* larval and pupal development.

## Introduction

Skeletor is the founding member of the *Drosophila* spindle matrix complex (reviewed in [1,2]) and localizes to interband regions of polytene chromosomes during interphase [3]. In order to determine other members of the complex interacting with Skeletor we have performed yeast two-hybrid interaction assays, and previously, we identified the chromodomain protein, Chromator, as such an interaction partner [3]. Another protein identified in this screen was a novel zinc-finger protein (CG14962) that we have named Digitor [4,5]. Recently, this protein was independently identified by sequence alignment in a search for orthologs of human ASCIZ by Zaytseva et al. [6] and referred to as dASCIZ by these workers. Digitor/dASCIZ has four zinc-finger domains at the amino-terminal end and a SQ/TQ cluster domain (SCD) with six TQT motifs in the carboxy-terminal domain [4,6]. TQT motifs have previously been identified in Dynein light chain binding proteins [7,8] and Digitor/dASCIZ has been shown to interact directly with Cut up, the DYNLL1 homolog in *Drosophila* [6].

By RNAi knock-down of Digitor/dASCIZ in developing wing discs Zaytseva et al. [6] demonstrated a role for Digitor/dASCIZ in regulating Dynein light chain expression as well as in mitotic progression. However, in order to determine global consequences of the absence of Digitor/dASCIZ during development in this study we have identified and characterized a P-element insertion that by qRT-PCR analysis appears to be a true null allele. Furthermore, we characterize the localization of Digitor/dASCIZ using a HA- and mCitrine-tagged Digitor/dASCIZ transgene. We provide evidence that Digitor/dASCIZ is a nuclear protein that performs multiple roles in *Drosophila* larval and pupal development.

## Materials and Methods

### *Drosophila melanogaster* stocks

Fly stocks were maintained at 25°C according to standard protocols [9] and Canton S was used for wild type preparations. The P element line *EP(3)3709* (w[1118]; P{w[+mC] = EP}Asciz[EP3709]/TM6B,Tb[1], RRID:BDSC_17158) and *Tubulin-mCherry* (w[*]; P{w[+mC] = UAS-ChRFP-Tub}2, RRID:BDSC_25774) were obtained from the Bloomington Drosophila Stock Center (NIH P400D018537). A full length Digitor construct (aa 1–388) was cloned into the *pPFHW* vector (Invitrogen) with a 3xHA tag in-frame at the NH_2_-terminus and an in-frame mCitrine tag at the COOH-terminal end using standard methods [10]. Transgenic *UAS*-*3xHA-Digitor-mCitrine* lines were generated by P-element-mediated transformation by BestGene. The expression of the transgene was driven using *tub-GAL4* (w[*]; P{w[+mC] = matalpha4-GAL-VP16}, RRID:BDSC_7062), *Act5C-Gal4* (y[1] w[*]; P{w[+mC] = Act5C-GAL4}17bFO1/TM6B, Tb[1], RRID:BDSC_3954), or *Sgs3-GAL4* (w[1118]; P{w[+mC] = Sgs3-GAL4.PD}TP1, RRID:BDSC_6870) drivers (Bloomington Drosophila Stock Center) introduced by standard genetic crosses. The fidelity of the construct was verified by sequencing at the Iowa State University DNA Facility. Viability assays were performed as in Zhang et al. [11]. Fly lines expressing a combination of transgenes were generated by standard genetic crosses. Balancer chromosomes and markers are described in Lindsley and Zimm [12]. For heat shock experiments, wandering third instar larvae were subjected to 50 min of heat shock treatment at 37°C as described previously [13].

### Identification and molecular characterization of Digitor

Skeletor cDNA sequence (*AF321290*) containing residues 215–474 was subcloned in-frame into the yeast two hybrid bait vector *pGBKT7* (Clontech) as previously described [3] using standard methods [10] and verified by sequencing (Iowa State University DNA Facility). The Skeletor bait was used to screen the Clontech Matchmaker 0–21 hr embryonic Canton-S yeast two-hybrid cDNA library as well as a *Drosophila* 0–2 hr embryonic yeast two-hybrid library (generous gift of Dr. L. Ambrosio, Iowa State University) as previously described [3,14]. Positive cDNA clones were isolated from both libraries, retransformed into yeast cells containing the Skeletor bait to verify the interaction, and sequenced. Homology searches identified the interacting clones as comprised of partial coding sequences from the *CG14962* locus.

### Antibodies

Primary antibodies used in this study include mouse anti-Skeletor [15], mouse anti-α-Tubulin (Sigma-Aldrich, T9026, RRID:AB_477593), rabbit anti-GFP (Invitrogen, CAB4211, RRID:AB_10709851), mouse anti-Pol IIo^ser5^ (Covance, MMS-134R, RRID:AB_10119940), mouse anti-HA (Roche, 11583816001, RRID:AB_514505), and rabbit anti-H3S10ph (Cell Signaling, #9701, RRID:AB_331535). The lamin Dm_0_ mAb HL1203 was provided by Drs. M. Paddy and H. Saumweber and has been previously characterized [16]. The mouse anti-BrdU mAb (RRID:AB_2618097) and the mouse anti-γH2Av mAb (RRID:AB_2618077) [17] were obtained from the Developmental Studies Hybridoma Bank.

### Biochemical Analysis

*SDS-PAGE and immunoblotting*. Protein extracts were prepared from third instar larvae homogenized in a buffer containing: 20 mM Tris-HCl pH 8.0, 150 mM NaCl, 10 mM EDTA, 1 mM EGTA, 0.2% Triton X-100, 0.2% NP-40, 2 mM Na_3_VO_4_, 1 mM PMSF, 1.5 μg/ml aprotinin as previously described [18]. Proteins were separated by SDS-PAGE and immunoblotted according to standard procedures [10]. For these experiments we used the Bio-Rad Mini PROTEAN III system, electroblotting to 0.2 μm nitrocellulose, and using anti-mouse or anti-rabbit HRP-conjugated secondary antibody (Bio-Rad) (1:3000) for visualization of primary antibody. Antibody labeling was visualized using chemiluminescent detection methods (SuperSignal West Pico Chemiluminescent Substrate, Pierce). The immunoblots were digitized using a ChemiDoc-It^®^TS2 Imager (UVP,LCC) and densitometric analysis was done using the built-in software.

*Pull-down experiments*. For *in vitro* pull down assays of Digitor/Skeletor interactions, a Skeletor cDNA fragment encoding residues 215–474 was subcloned in frame into the *pET28a* vector (Novagen) containing 6x His-tags. A Digitor cDNA fragment corresponding to amino acid residues 144 to 376 without the NH_2_-terminal zinc finger domains was subcloned in-frame into the *pGEX-4T3* vector (Amersham Biosciences) with a GST-tag. As a control construct the amino-terminal fragment of the JIL-1 kinase [19,20] corresponding to the first 260 amino acid residues was subcloned in frame into the *pET28a* vector. The tagged protein constructs were expressed in BL21 cells (Novagen). For His pull-down assays, approximately 10 μg of His-Skeletor or His-JIL-1 were coupled with Ni-NTA beads (Qiagen) and incubated with 10 μg of GST-Digitor in 1X PBS (pH 7.4) for 4 hr at 4°C. The protein complex-coupled beads were given a total of 5 washes of 20 min each (2 washes with 0.04% PBST, 2 washes with 0.02% PBST, and a final wash in 1X PBS) and analyzed by SDS-PAGE and immunoblotting using anti-GST mAb 8C7 [3]. Similarly for GST pulldown assays, GST-Digitor or GST alone was bound to glutathione agarose beads (Sigma) and incubated with His-tagged Digitor in 1X PBS (pH 7.4) for 4 hr at 4°C. The protein complex coupled beads were given a total of 5 washes of 20 min each (2 washes with 0.04% PBST, 2 washes with 0.02% PBST, and a final wash in 1X PBS) and analyzed by SDS-PAGE and immunoblotting using anti-His antibody (Boehringer Mannheim).

For *in vitro* pull-down assays of Digitor/Cut up interactions, a *Digitor* cDNA fragment encoding amino acids 256 to 388 was subcloned in-frame into the *pET28a* vector and a full-length *Cut-up* cDNA fragment encoding amino acids 1–89 was subcloned into the *pMAL-C2X* vector (New England BioLabs) with a MBP tag. The tagged protein constructs were expressed in BL21 cells. For His pull-down assays, approximately 10 μg of His-Digitor or His-Skeletor were coupled with Ni-NTA beads (Qiagen) and incubated with 10 μg of MBP-Cut-up in 1X PBS (pH 7.4) for 4 hr at 4°C. The protein complex coupled beads were given a total of 5 washes of 20 min each (2 washes with 0.04% PBST, 2 washes with 0.02% PBST, and a final wash in 1X PBS) and analyzed by SDS-PAGE and immunoblotting using anti-MBP antibody (New England BioLabs). Similarly for the MBP pulldown assays, MBP-Cut up or MBP alone was bound to amylose resin (New England BioLabs) and incubated with His-tagged Digitor 1X PBS (pH 7.4) for 4 hours at 4°C. The protein complex-coupled beads were given a total of 5 washes of 20 min each (2 washes with 0.04% PBST, 2 washes with 0.02% PBST, and a final wash in 1X PBS) and analyzed by SDS-PAGE and immunoblotting using anti-His antibody (Boehringer Mannheim).

### Immunohistochemistry

Standard polytene chromosome squash preparations were performed as in Cai et al. [21] using either 1 or 5 minute fixation protocols and labeled with antibody as described in Jin et al. [19]. S2 cell immunocytochemistry using 4% paraformaldehyde fixation protocols were performed as described in Qi et al. [22]. Immunocytochemistry for H3S10ph in dissected third instar larval brains using 4% paraformaldehyde fixation was performed according to the protocol of Wu and Luo [23] and Acridine Orange labeling of third instar larval brains was performed as in Sullivan et al. [24]. BrdU labeling of third instar larval brains was performed essentially as in Truman et al. [25] and in Bolkan et al. [26]. In brief, dissected brains were incubated in Shields and Sang M3 insect medium (Sigma) containing 1 mg/ml 5-bromo 2-deoxyuridine (BrdU; Sigma) for 1 hr. The brains were then fixed in 4% paraformaldehyde, permeabilized in 0.04% PBST, and treated with 2N HCl in PBST for 30 min at room temperature. After a series of washes BrdU incorporation was detected by performing immunocytochemistry. DNA was visualized by staining with Hoechst 33258 (Molecular Probes) in PBS. The appropriate species- and isotype- specific TRITC-, and FITC-conjugated secondary antibodies (Cappel/ICN, Southern Biotech, Jackson Immuno Research) were used (1:200 dilution) to visualize primary antibody labeling. All antibody-labeling washes were repeated 3 times for 10 min each with PBST (PBS + 0.2% Triton X-100). As previously described [22] the final preparations were mounted in 90% glycerol containing 0.5% n-propyl gallate and examined using epifluorescence optics on a Zeiss Axioskop microscope and images were captured and digitized using a cooled Spot CCD camera. Images were imported into Photoshop where they were pseudocolored, image processed, and merged. In some images non-linear adjustments were made to the channel with Hoechst labeling for optimal visualization of chromosomes.

### Expression of tagged constructs in S2 cells

A full-length Digitor cDNA was cloned into the *pMT/V5-HisA* vector (Invitrogen) with an in-frame GFP tag at the COOH-terminus using standard methods [10]. Additionally, a full-length Cut up construct with an mCherry tag and a full-length H2Av construct with a GFP tag were generated in the *pAFHW* vector (Invitrogen). The fidelity of all constructs was verified by sequencing at the Iowa State University DNA Facility. As previously described [22] *Drosophila* Schneider 2 (S2) cells were cultured in Shields and Sang M3 insect medium (Sigma) supplemented with 10% fetal bovine serum, antibiotics and L-Glutamine at 25°C. The S2 cells were transfected with the different tagged constructs or in combination using a calcium phosphate transfection kit (Invitrogen). Expression was either constitutive (Cut up-mCherry and H2Av-GFP) or induced by 0.5 mM CuSO_4_ (Digitor-GFP). Cells expressing the constructs were harvested 18–24 h after induction and affixed onto Concavalin-A coated coverslips for immunostaining and Hoechst labeling or images of the fluorescently tagged constructs were captured live and digitized using a cooled Spot CCD camera.

### Timelapse confocal microscopy

Timelapse imaging of the fluorescently-tagged constructs in live syncytial embryos was performed using a Leica TCS SP5 tandem scanning microscope as previously described in Ding et al. [27] and in Yao et al. [28]. In short, 0–1.5 h embryos were collected from apple juice plates, and aged 1 h. The embryos were manually dechorionated, transferred onto a cover slip coated with a thin layer of heptane glue, and covered with a drop of Halocarbon oil 700. Timelapse image sequences of a single z-plane or of z-stacks covering the depth of the mitotic apparatus were obtained using a Plan-apochromat 63X 1.4 NA objective.

### Phenotypic analysis

Wild-type and *Digitor* mutant pupae were synchronized at the white prepupal stage (T = 0) and incubated at 25°C for either 24 hr, 90 hr, or 168 hr in a humidified incubator to document development during metamorphosis. Images of the pupae were obtained using a cooled Spot CCD camera. Analysis of pupal developmental stages was done in accordance with Bainbridge and Bownes [29]. For analysis of the relative brain size of wild-type compared to the *Digitor* mutant, third instar larvae brains were dissected in PBS and imaged using a cooled Spot CCD camera. The area of the two optic lobes from wild-type and mutant larvae was determined using Image J software (NIH) and results compared using a Student's two-tailed t-test. To compare wild-type and *Digitor* mutant third instar larval body size, 22 larvae of each genotype were weighed on an analytical balance. Four replicates for each genotype were compared using a Student's two-tailed t-test. For analysis of the relative BrdU incorporation and H3S10ph antibody-labeling in wild-type and *Digitor* mutant third instar larval brains we obtained images of the fluorescently labeled preparations using a cooled Spot CCD camera. The area of the two optic lobes and thoracic segments from wild-type and mutant larvae was outlined and average pixel intensity of the fluorescence determined using the Image J software (NIH). Background fluorescence was determined in the abdominal segments and subtracted. The results were compared using a Student's two-tailed t-test.

### RNA isolation and gene expression analysis

RNA was isolated from wandering third instar larvae or staged pupae using UltraClean® tissue and cells RNA isolation kit (MO BIO Laboratories, Inc.) according to the manufacturer’s instructions. 1 μg of RNA was DNAse (Invitrogen) treated and converted into the first strand of cDNA using the RT-PCR SuperScript III kit (Invitrogen) according to the manufacturer’s instructions. For determining changes in gene expression quantitative PCR was carried out with the cDNA template using Brilliant® II SYBR Green QPCR Master Mix (Stratagene) in an Mx4000 PCR machine (Stratagene). Cycling parameters used were 10 min at 95°C, followed by 40 cycles of 30 s at 95°C, 30 s at 55°C, and 30 s at 72°C. Fluorescence intensities were plotted against the number of cycles using an algorithm provided by Stratagene. Relative abundance in transcripts was calculated according to the 2-ΔΔCt method [30].

For analysis of changes in ecdysone signaling in staged pupae, RT-PCR was conducted as follows: total RNA was isolated from whole wild-type and *EP(3)3709/EP(3)3709* staged prepupae and pupae at designated time-points. 1 μg of RNA was converted into cDNA and analyzed by RT-PCR. Digital images of the RT-PCR products after agarose gel separation containing ethidium bromide [10] were obtained with a SONY CCD camera and densitometric analysis performed using Image J software (NIH). Relative level of expression was determined by normalizing average pixel intensity of the experimental bands to the average pixel intensity of *RP49* bands. Thermocycler conditions used were as follows: initial denaturation at 94°C for 5 min followed by 94°C for 1 min, 60°C for 1min, 72°C for 1 min, and a final 10 min 72°C elongation period. The cycle numbers used varied from 22–35 depending on the individual genes and primer pairs analyzed. The primer pairs used in this study are listed in S1 Table. For RT-PCR analysis, results were obtained from three independent biological replicates.

### Paraquat induced stress test

A paraquat resistance test was performed essentially as described in Vermeulen et al. [31] and in Bonilla et al. [32]. In brief, for each genotype, a total of 50 adult flies were assayed per sex. Virgin flies from both wild-type and *Digitor* heterozygous mutant backgrounds from a single egg batch grown under non-crowding conditions were collected within the same day, sex-segregated, and aged in standard food-containing vials for 5 d at 25°C. The aged flies were then starved for 6 hr by transferring them into vials containing only water soaked filter papers to avoid desiccation effects and to also ensure that no food remained in their digestive tract at the end of the starvation period. Afterwards, the flies were transferred to vials containing filter paper soaked with 20 mM paraquat in a 5% sucrose solution. Resistance was scored as percent survival at 12 hr intervals for a total of 48 hr after the start of paraquat exposure. Five independent replicates were conducted for each experimental condition.

## Results

### The spindle matrix protein Skeletor interacts with Digitor/dASCIZ

In order to identify candidates for proteins comprising the spindle matrix macromolecular complex (reviewed in [1,2]) we conducted yeast two-hybrid interaction assays using a Skeletor bait construct containing amino acids 215 through 474 that alone was unable to activate transcription of the reporter genes [3]. Two different embryonic yeast two-hybrid libraries were screened (0–2 hr and 0–21 hr) and interacting clones from both libraries comprising *CG14962* coding sequences were identified. The *CG14962* locus is located on the third chromosome and has two alternative transcripts due to variant use of different 5' exons; however, the coding sequence is identical and without introns for both transcripts (Flybase). The resulting protein has four zinc-finger domains at the amino-terminal end and a SCD with six TQT motifs in the carboxy-terminal domain and has homology to human ASCIZ (ATM substrate Chk2-interacting Zn^2+^ finger) that was originally identified as a stress-response protein involved in signaling induced by DNA damage [31]. Due to the zinc-finger domains *CG14962* was named *Digitor* by Sengupta et al. [4,5] and *dASCIZ* by Zaytseva et al. [6] based on its homology. Analysis of the isolated Digitor/dASCIZ yeast two-hybrid library clones suggested that the interaction region with Skeletor was COOH-terminally located and independent of the zinc-finger region.

**Fig 1:**
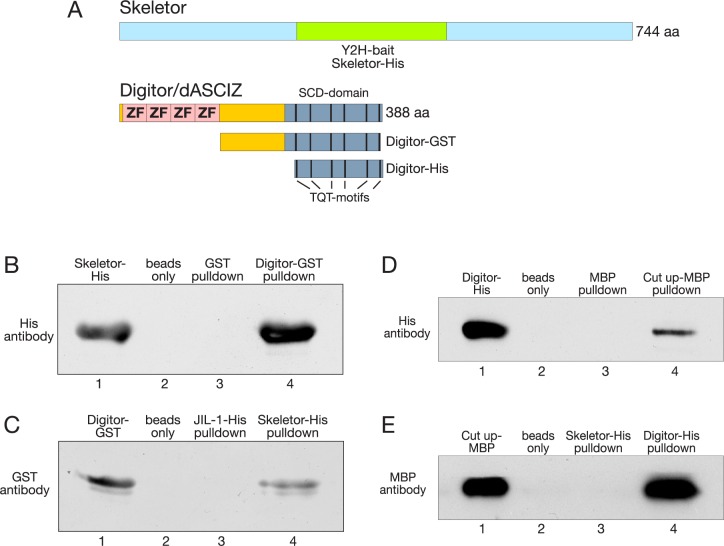
Digitor/dASCIZ interact with Skeletor and Cut up in pull down assays. (A) Diagrams of full-length Skeletor and Digitor/dASCIZ and the fragments used for yeast two-hybrid assays. The region of Skeletor corresponding to the yeast two-hybrid (Y2H) bait and Skeletor-His is indicated in green. Full-length Digitor/dASCIZ has four zinc-finger domains (ZN) and a SCD with six TQT-motifs and the two GST- and His-tagged COOH-terminal fragments, respectively, are indicated below. (B) A Digitor-GST construct pulls down Skeletor-His as detected by His antibody (lane 4). Beads-only and GST-only pulldown controls were negative (lane 2 and 3). Lane 1 shows the position of the Skeletor-His fusion protein. (C) A Skeletor-His construct pulls down Digitor-GST as detected by GST antibody (lane 4). Beads only and JIL-1-His pulldown controls were negative (lane 2 and 3). Lane 1 shows the position of the Digitor-GST fusion protein. (D) A Cut up-MBP construct pulls down Digitor-His as detected by His antibody (lane 4). Beads only and MBP only pulldown controls were negative (lane 2 and 3). Lane 1 shows the position of the Digitor-His fusion protein. (E) A Digitor-His construct pulls down Cut up-MBP as detected by MBP antibody (lane 4). Beads-only and Skeletor-His pulldown controls were negative (lane 2 and 3). Lane 1 shows the position of the Cut up-MBP fusion protein.

To confirm the physical interaction of Digitor/dASCIZ with Skeletor, we performed *in vitro* pull down experiments using a His-tagged Skeletor fusion protein containing the yeast two-hybrid bait region and a Digitor-GST fusion protein without the four zinc-finger motifs (Fig 1A). Whereas GST protein alone or beads only were not able to pull down Skeletor, the Digitor-GST fusion protein pulled down a band corresponding to the size of Skeletor-His that was detected by His-antibody (Fig 1B). In the reverse experiment, Skeletor-His fusion protein was able to pull down Digitor-GST as detected by GST-antibody (Fig 1C). However, beads only and a His-tagged JIL-1 kinase control fusion protein did not (Fig 1C). These results support the existence of a direct physical interaction between Skeletor and Digitor/dASCIZ that does not require the zinc-finger region.

The presence of the TQT motifs in Digitor/dASCIZ indicates an interaction with the Dynein light chain as has been previously reported for Cut up in *Drosophila* by Zaytseva et al. [6]. We provide further evidence for this interaction by *in vitro* pulldown assays with a His-tagged COOH-terminal fragment of Digitor containing the six TQT motifs (Fig 1A) and a full length Cut up MBP fusion protein. As illustrated in Fig 1D whereas MBP protein alone or beads only were not able to pull down Digitor, the Cut up-MBP fusion protein pulled down a band corresponding to the size of Digitor-His that was also detected by His-antibody. In the reverse experiment, Digitor-His fusion protein was able to pull down Cut up-MBP as detected by MBP-antibody (Fig 1E). However, beads only and a His-tagged Skeletor control fusion protein did not (Fig 1E).

### Digitor/dASCIZ is localized to the nucleus at interphase and the spindle matrix during mitosis

**Fig 2:**
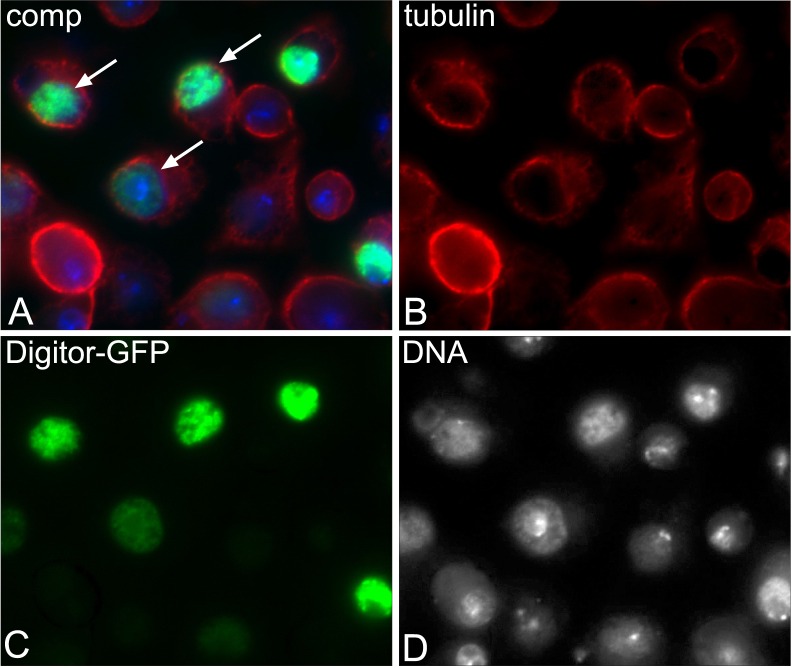
Digitor/dASCIZ localizes to the nucleus. (A-D) S2 cells transiently transfected with a GFP-tagged Digitor construct were fixed and labeled with antibodies to GFP (in green), tubulin (in red), and Hoechst for DNA (in blue/grey). In the composite image (A) the nuclear localization of Digitor-GFP is indicated by arrows.

**Fig 3:**
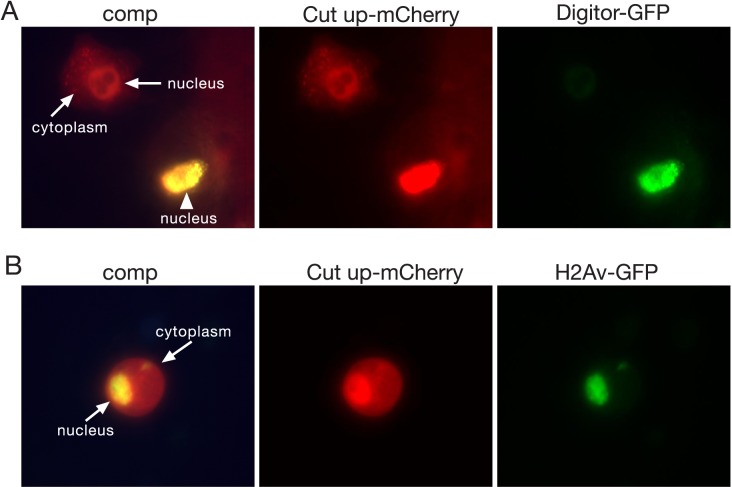
Live imaging of transiently transfected S2 cells with Digitor-GFP and Cut up-mCherry. (A) Co-transfection of Cut up-mCherry together with Digitor-GFP. Cut up-mCherry in singly transfected cells was localized both to the nucleus and the cytoplasm (arrows); however, in co-transfected cells, Cut up-mCherry was found only in the nucleus together with Digitor-GFP (Arrowhead). (B) In cells co-transfected with Cut up-mCherry and an H2Av-GFP control construct, Cut up-mCherry retained its dual localization to both the nucleus and cytoplasm (arrows).

In order to study the cellular localization of Digitor we performed transient expression studies of a full-length GFP-tagged Digitor construct in *Drosophila* S2 cells. As illustrated in Fig 2 Digitor-GFP localized exclusively to the nucleus of transfected cells (221 out of 221 cells, 100%). To further document the interaction with Cut up we co-transfected S2 cells with Digitor-GFP and Cut up-mCherry (Fig 3). Cut up-mCherry in singly transfected cells was localized both to the nucleus and in the cytoplasm (241 out of 241, 100%). However, in 73 out of 235 (31%) co-transfected cells, Cut up-mCherry was only found in the nucleus together with Digitor-GFP (Fig 3A). In cells co-transfected with Cut up-mCherry and an H2Av-GFP control construct, Cut up-mCherry retained its dual localization to both the nucleus and cytoplasm (210 out of 210 cells examined) (Fig 3B). Taken together these data suggest that overexpressed Digitor-GFP binds and recruits Cut up-mCherry to the nucleus providing evidence for an *in vivo* interaction between Digitor/dASCIZ and Cut up.

**Fig 4:**
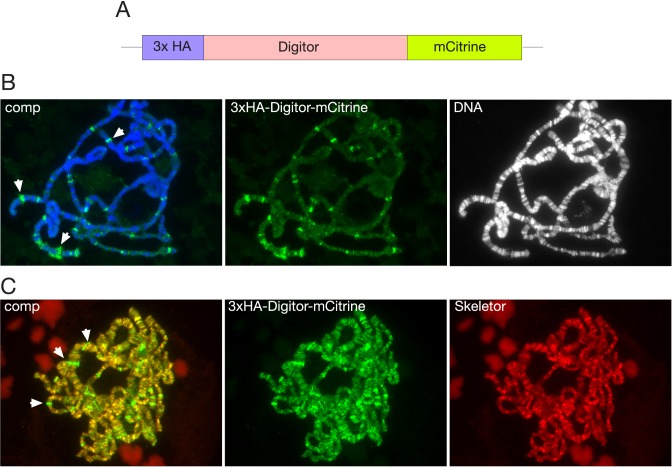
Transgenic expression of 3xHA-Digitor-mCitrine in salivary gland cells. (A) Diagram of 3xHA-Digitor-mCitrine. (B) Polytene squash preparation labeled with anti-HA antibody to detect the 3xHA-Digitor-mCitrine construct (in green) and with Hoechst to label DNA (in blue/grey). In the composite image (comp) examples of developmental puff regions with higher expression of 3xHA-Digitor-mCitrine are indicated by arrowheads. (C) Polytene squash preparation labeled with anti-HA antibody to detect the 3xHA-Digitor-mCitrine construct (in green) and with anti-Skeletor antibody (in red). In the composite image (comp) co-localization between 3xHA-Digitor-mCitrine and Skeletor is indicated by yellow/orange colors. Examples of developmental puff regions positive for 3xHA-Digitor-mCitrine but not for Skeletor are indicated by arrowheads. The relatively low exposure of anti-HA antibody-labeling of 3xHA-Digitor-mCitrine in (B) shows the prominent labeling of the developmental puffs whereas the longer exposure in (C) highlights the labeling of the weaker labeled interband regions.

**Fig 5:**
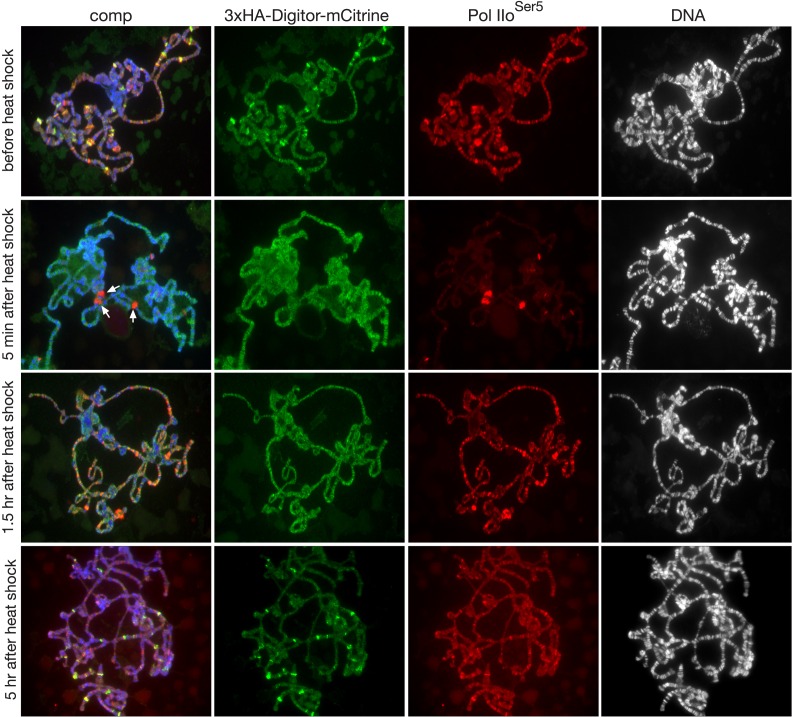
Localization of 3xHA-Digitor-mCitrine before and after heat shock treatment. The figure shows images of polytene squash preparations before heat shock as well as 5 min, 1.5 hr, and 5 hr after heat shock. The preparations were labeled with anti-HA antibody to detect the 3xHA-Digitor-mCitrine construct (in green), with anti-Pol IIo^ser5^ antibody (in red), and with Hoechst for DNA (in blue/grey). Arrows indicate Pol IIo^ser5^-positive heat shock-induced puffs 5 min after treatment.

To further explore the nature of the nuclear localization of Digitor/dASCIZ we expressed a *3xHA-Digitor-mCitrine* full length construct (Fig 4A) transgenically in third instar larvae under *UAS-Gal4* control. Under non-stressed conditions when driven by the *sgs3-Gal4* driver, 3xHA-Digitor-mCitrine binds to polytene chromosomes at interband regions in a complementary pattern to that of Hoechst (Fig 4B) and co-localizes with Skeletor (Fig 4C). Interestingly, enhanced levels of 3xHA-Digitor-mCitrine but not of Skeletor at developmental puff regions [33] was especially prominent (Fig 4B and 4C). Furthermore, many but not all of these puffs were also labeled by antibody to the paused form of RNA Polymerase II (Pol IIo^ser5^) (Fig 5). This pattern of labeling is compatible with a general role for Digitor/dASCIZ as a transcriptional regulator of development. Since human ASCIZ is known to be a stress response protein [34,35] we performed heat shock experiments on third instar salivary glands (Fig 5) in order to examine whether this pattern changes under stressed conditions. Polytene chromosome squashes were made from salivary glands at control conditions (before heat shock) as well as 5 min, 1.5 hr, and 5 hr after heat shock. The preparations were double labeled with Pol IIo^ser5^ a marker for heat shock puff regions [36]. In response to heat shock treatment there is a redistribution of Pol IIo^ser5^ labeling which is reduced at most sites, while being dramatically upregulated at heat shock puffs where transcription of heat shock-activated genes is occurring [36] (Fig 5). In contrast, 3xHA-Digitor-mCitrine was released only from developmental puff regions and was not recruited to heat-shock induced puffs (Fig 5). While the non heat-shock pattern of Pol IIo^ser5^ labeling was restored after 1.5 hr the binding of 3xHA-Digitor-mCitrine to developmental puff regions recovered on a slower time scale and was only restored 5 hr after heat-shock (Fig 5). This dynamic relocation of Digitor/dASCIZ from developmental puff regions during heat-shock conditions is compatible with the hypothesis that Digitor/dASCIZ may modulate gene expression at these sites and that this modulation changes during the stress response.

**Fig 6:**
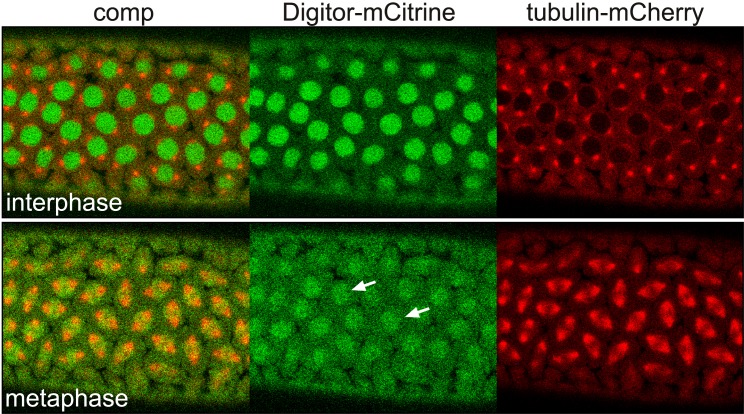
Confocal time-lapse analysis of 3xHA-Digitor-mCitrine during mitosis in a syncytial *Drosophila* embryo. The figure shows the relative dynamics of the 3xHA-Digitor-mCitrine construct (in green) and tubulin-mCherry (in red) at interphase and metaphase, respectively. Arrows indicate the localization of the 3xHA-Digitor-mCitrine construct in the spindle region after NEB and microtubule spindle formation at metaphase.

The interaction of Digitor/dASCIZ with Skeletor suggest a possible interaction with the spindle matrix during cell division. To explore this possibility we performed time-lapse imaging of 3xHA-Digitor-mCitrine together with tubulin-mCherry during mitosis in syncytial *Drosophila* embryos. The results showed (Fig 6 and S1 Movie) that a considerable fraction of 3xHA-Digitor-mCitrine remains in the nuclear space after nuclear envelope breakdown (NEB) embedding the forming microtubule spindle apparatus, a defining feature of spindle matrix proteins [28]. After NEB there are no diffusion barriers even for greater than 2000 kDa molecules [28] suggesting that Digitor/dASCIZ's confinement to the spindle region during mitosis is mediated by molecular interactions with the spindle matrix complex. A likely mechanism for such interactions would be Digitor/dASCIZ's direct physical association with Skeletor.

### Digitor/dASCIZ is an essential protein

**Fig 7:**
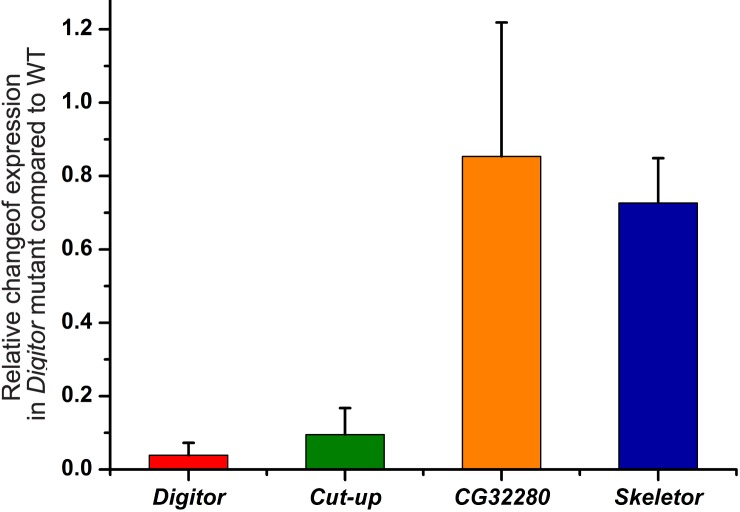
Transcript levels in the *Digitor* null mutant relative to wild type. Relative transcript levels of *Digitor/dASCIZ*, *Cut up*, *CG32280*, and *Skeletor* in *Digitor* mutant (*EP(3)3709/EP(3)3709*) third instar larvae as compared to wild type. The transcript levels of all four genes were determined by qRT-PCR and fold change was calculated according to the 2-ΔΔCt method. Each determination was performed in triplicate and the average is shown with S.D.

In order to determine the phenotypic consequences of the absence of Digitor/dASCIZ during development we have identified a P element, *EP(3)3709*, inserted 27 bp downstream of the *Digitor/dASCIZ* ATG start codon that is likely to be a true null allele. To verify this we performed qRT-PCR of transcripts from *Digitor/dASCIZ*, *Cut up*, *Skeletor*, and the neighboring gene *CG32280* extracted from Wild-type and *EP(3)3709* homozygous third instar larvae, respectively. The Dynein light chain gene *Dynll1* (*Cut up* in *Drosophila*) has been established to be an evolutionarily conserved transcriptional target of ASCIZ proteins down regulating its expression [6,37]. As illustrated in Fig 7 the level of *Digitor/dASCIZ* and *Cut up* transcripts were severely reduced in the mutant compared to wild-type whereas the transcript level of *CG32280* and *Skeletor* was largely unaffected. The low remaining transcript level of *Digitor/dASCIZ* in the mutant is likely to be remnants from maternal stores. The *EP(3)3709* P element line is homozygous lethal at pupal stages. Of 113 *EP(3)3709* homozygous larvae examined, all pupated but none eclosed. In contrast, of 120 wild-type larvae the eclosion rate was 94% and of 125 heterozygous *EP(3)3709* larvae the eclosion rate was 87%.

To verify that the observed lethality was caused by impaired Digitor/dASCIZ function we used *3xHA-Digitor-mCitrine* as a rescue construct using a *Act5C-Gal4* driver line. Thus, in the following cross: *3xHA-Digitor-mCitrine*, *w+/CyO*, *y+; EP(3)3709/TM6b*, *Sb*,*Tb*, *e*^*s*^ X *Act5C-Gal4*, *w+*/*CyO*,*y+; EP(3)3709*/*TM6b*, *Sb*, *Tb*, *e*^*s*^ rescue would be indicated by the presence of adult progeny that lack the *Sb* phenotype. Out of 249 adult flies examined from such a cross we found 26 flies with the non-*Sb* phenotype (S2 Table). This indicates that the 3xHA-Digitor-mCitrine construct can provide partial rescue function (31%) supporting that the *EP(3)3709* is a null or strong hypomorphic *Digitor/dASCIZ* allele and that *Digitor/dASCIZ* is an essential gene.

**Fig 8:**
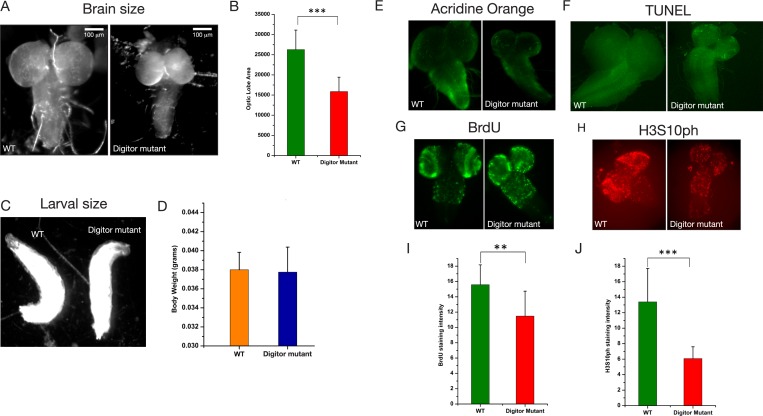
*Digitor* mutant third instar larvae have a severely reduced brain size. (A) Images of dissected brains from wild type (WT) and *Digitor* mutant (*EP(3)3709/EP(3)3709*) third instar larvae at the same scale. (B) Comparison of the average area of the two optic lobes from 20 brains of wild type and *Digitor* mutant larvae with S.D., respectively. The difference in size was statistically significant (P value < 0.001, Student's two-tailed t-test). (C) Images of wild type and *Digitor* mutant third instar larvae side by side. (D) Comparison of the average from four determinations of the body weight of 22 wild type and *Digitor* mutant larvae with S.D., respectively. The difference in weight was not statistically significant (P value ≥ 0.8, Student's two-tailed t-test). (E) Acridine Orange labeling of brains from wild type and *Digitor* mutant larvae. (F) TUNEL labeling of brains from wild type and *Digitor* mutant larvae. (G) BrdU incorporation into brains from wild type and *Digitor* mutant larvae. (H) Anti-H3S10ph antibody labeling of brains from wild type and *Digitor* mutant larvae. (I) Comparison of the average fluorescence intensity of BrdU incorporation from the two optic lobes and thoracic segments from 10 brains of wild type and *Digitor* mutant larvae with S.D., respectively. The difference was statistically significant (P value < 0.01, Student's two-tailed t-test). (J) Comparison of the average fluorescence intensity of anti-H3S10ph antibody labeling from the two optic lobes and thoracic segments from 8 brains of wild type and *Digitor* mutant larvae with S.D., respectively. The difference was statistically significant (P value < 0.001, Student's two-tailed t-test).

The enrichment and localization of Digitor/dASCIZ to developmental puff regions suggest a role in organ maturation and metamorphosis. An early phenotype we observed in homozygous *EP(3)3709* (*Digitor* mutant) crawling third instar larvae was a severely reduced brain size (Fig 8A and 8B). We quantified this aspect by comparing the area of the two optic lobes of brains from Wild-type and *Digitor* mutant larvae, respectively. As illustrated in Fig 8B there was a statistically significant (p<0.001) reduction of this parameter in *Digitor* mutant larvae to about half that of Wild-type larvae. Interestingly, this was not a consequence of a general decline in larval development as the size and weight of *Digitor* mutant larvae were indistinguishable from that of Wild-type larvae (Fig 8C and 8D). A small brain phenotype could be the result of decreased cell proliferation, increased cell death, or a combination of both. To distinguish between these scenarios we first assessed the levels of cell death and apoptosis in brains from *Digitor* mutant and Wild-type larvae by Acridine Orange (Fig 8E) and TUNEL (Fig 8F) labeling. The results showed that compared to Wild-type there was only a slight increase in cell death in the *Digitor* mutant that was not likely to be able to account for the markedly decreased brain size. However, both BrdU labeling, a marker for actively replicating cells [26] and H3S10ph labeling, a marker for cells in mitosis [38] showed significantly fewer cells in the actively dividing proliferation brain zones (Fig 8G–8J) in the *Digitor* mutant. Taken together these results suggest that impaired cell proliferation was the main cause of the mutant small brain phenotype.

### Digitor/dASCIZ is required for metamorphosis

**Fig 9:**
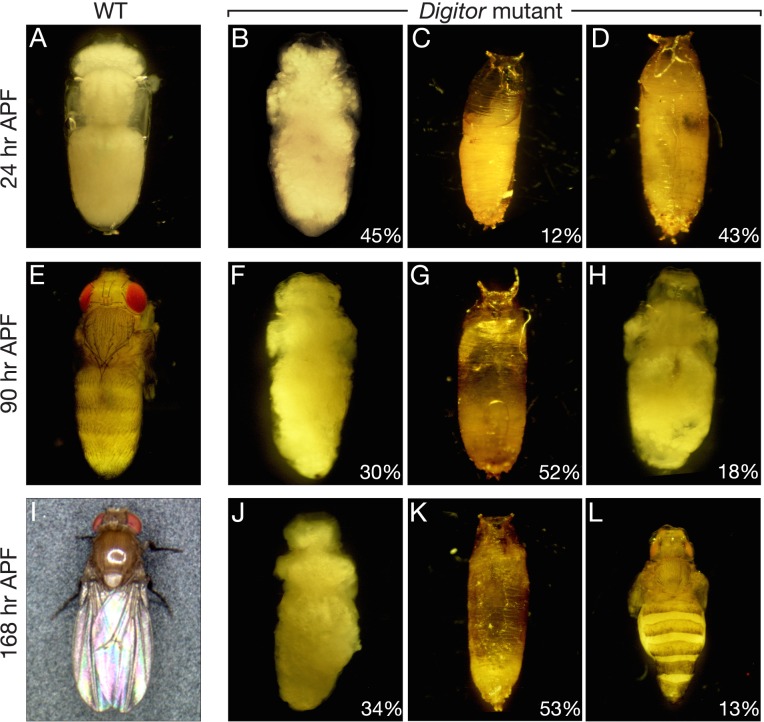
Defective metamorphosis of *Digitor* mutant pupae. *Digitor* mutant (*EP(3)3709/EP(3)3709*) and wild type pupae were selected as white prepupae and monitored through prepupal and pupal development 24, 90, and 168 hr after puparium formation (APF). (A) Wild type pupae 24 hr APF. (B-D) Examples of *Digitor* mutant pupae with impaired and/or defective development at 24 hr APF. (E-H) Wild type and *Digitor* mutant pupae with impaired or defective metamorphosis at 90 hr APF. (I) At 168 hr APF wild type pupae had eclosed as adult flies. (J-L) *Digitor* mutant pupae all failed to eclose at 168 hr APF and were arrested at various stages of pupal development. The frequency of different types of *Digitor* mutant pupae is indicated in percent. In (A), (B), (E), (F), (H), (J), and (L) the pupal case was removed.

*Digitor* mutants are pupal lethals, however, the exact time at which lethality occurs is not precise and varies from very early metamorphosis with the formation of a brown puparium with no detectable differentiation of tissue, to late metamorphosis with dead pharate adults. To further analyze the contribution of Digitor/dASCIZ in metamorphosis, *Digitor* mutants were selected as white prepupae and monitored through prepupal and pupal development and analyzed as in Bainbridge and Bownes [29]. At 24 hours after puparium formation (APF), the wild-type population showed posteriorly displaced imagos with proper imaginal disc eversion, and legs and wings were fully extended along the abdomen (Fig 9A). Gas bubbles could be clearly detected in the anterior end whereas salivary glands were completely degenerated and green malphigian tubules were visible in the abdomen (Fig 9A). At this time-point, most of the *Digitor* mutant population was either decayed or undifferentiated without proper head or imaginal disc eversion (Fig 9C and 9D). In some cases within the pupal case the gas bubble was still present posteriorly displacing the imago anteriorly. In the remaining 45% of the mutant population that was not desiccated and had the correct gas bubble displacement, formation of adult structures was impaired (Fig 9B). In these pupae the imaginal discs everted normally and the clear pupal cuticle was formed, but within this cuticle the integrity of the epithelial sheets in the imaginal tissues was severely compromised (Fig 9B). An ecdysone pulse approximately at 10–12 hr post puparium formation normally triggers programmed cell death of the larval salivary glands [39,40]. To determine if this response is defective in the *Digitor* mutant, presence or absence of salivary glands was analyzed in mutant pupae at 24 hr APF. The results showed that salivary glands still could be detected in 12 out of 13 mutant animals analyzed within the 45% mutant pupae population that was not desiccated and had the correct gas bubble displacement suggesting a defective ecdysone response.

At 90 hours APF, the wild-type population showed bright red pigmentation in the eyes with the full complement of bristles; the abdomen was segmented and developed; wings were folded and almost black in color (Fig 9E). In contrast, at this time point, 52% of the mutant population appeared shriveled and decayed (Fig 9G). Another 30% of the population had impaired development comparable to the 24 hr APF mutants (Fig 9F). In the remaining 18% of the population some rudimentary development of adult organs and appendages could be detected (Fig 9H).

After 96 hours APF the wild-type population eclosed as adult flies (Fig 9I); however, there were no eclosion events observed within the *Digitor* mutant population, which was analyzed for another 72 hr. At 168 hr APF, 53% of the mutant population were shriveled, decayed and degenerated (Fig 9K), and another 33% of the population were without distinct adult organs inside the pupal cuticle (Fig 9J). Interestingly, in a small population (13%) further signs of development compared to the 96 hr APF-mutants could be detected with pigmentation of the eyes along with bristles in head, thorax, legs and abdomen (Fig 9L). However, the different populations of mutant pupae described all failed to eclose with no escapers and eventually degenerated. Taken together, these results suggest a critical role for *Digitor/dASCIZ* during metamorphosis.

### Defective ecdysone signaling in the *Digitor* mutants during early metamorphosis

A major regulator of metamorphosis and subsequent eclosion is the ecdysone-signaling cascade that is tightly controlled by the expression of ecdysone-regulated genes [41,42]. Defective ecdysone signaling caused by an aberrant expression of one or more genes of the signaling cascade has been implicated in impaired metamorphosis in numerous studies [39,43–50]. Analysis of the pupal phenotypes in the *Digitor* mutant raised the possibility that these phenotypes were triggered by changes in expression of key ecdysone-regulated genes. To test this hypothesis, we analyzed the expression of a set of ecdysone-regulated genes, namely, *ECR*, *E74*, *BR-C*, *βFTZ-F1*, and *E93* at 3 time-points: at white puparium formation (T_0_), at the mid-prepupal period (T_8_ measured as 8 hr after T_0_), and at the time just after head eversion (T_14_ measured as 14 hr after T_0_).

**Fig 10:**
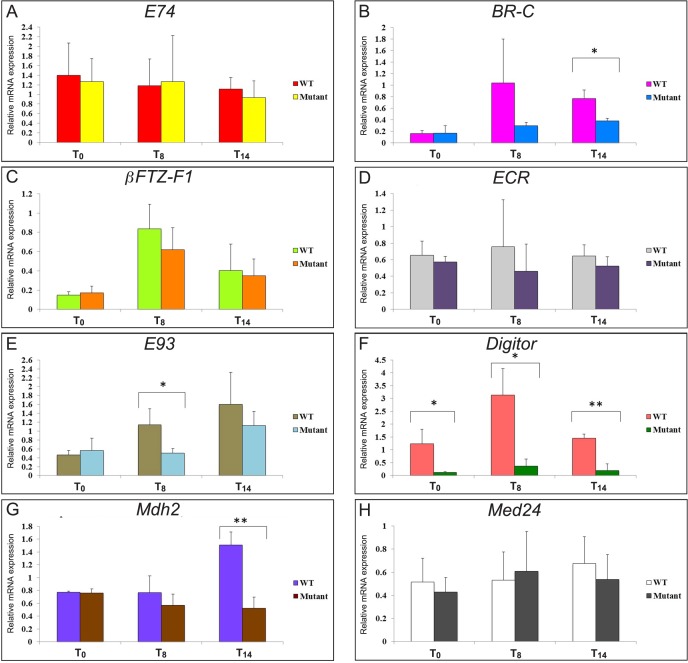
Comparison of mRNA expression of ecdysone-regulated and other genes relevant for morphogenesis in wild type and *Digitor* mutant prepupae and pupae. Relative levels of *E74* (A), *BR-C* (B), *βFTZ-F1 (*C), *ECR* (D), *E93* (E), *Digitor* (F), *Mdh2* (G), and *Med24* (H) mRNA expression were determined at the white prepupae stage (T_0_), mid-prepupal stage (T_8_), and just after head eversion (T_14_) and normalized to *RP49* expression levels. Each bar represents the average of three independent replicates with S.D. Statistically significant expression differences were determined using a Student's two-tailed t-test (*, P < 0.05; **, P < 0.01).

In the experiments RNA was isolated from white prepupae (T_0_), mid prepupae (T_8_) and early pupae (T_14_) from both wild-type and *Digitor* mutant backgrounds, converted into cDNA and analyzed for the expression of the aforementioned genes by RT-PCR (Fig 10)^.^. All experiments were performed in triplicate. Digitor/*dASCIZ* expression was significantly reduced in the mutants at all time points examined (Fig 10F). Many of the ecdysone-regulated transcripts surveyed registered a slight decrease in abundance relative to wild-type at both T_8_ and T_14_ (Fig 10); however, only the expression of *BR-C* and *E93* at T_8_ and *BR-C* and *Mdh2* at T_14_ were significantly reduced (Fig 10B, 10E, and 10G) with likely consequences for development. *BR-C* is a key ecdysone inducible gene required for metamorphosis and has been shown to perform a plethora of functions including morphogenesis of imaginal discs to form continuous and compact adult structures [46,51,52]. Hence downregulation of *BR-C* may account for many of the observed defects and general lack of development in *Digitor* mutant pupae. The expression of *E93* was significantly decreased at T_8_ but not at T_14_ (Fig 10E). Previous studies have shown that *E93* is induced in the larval midgut at the mid prepupae stage catalyzing the destruction of midgut gastric caeca and midgut shortening [39]. Thus reduced expression of *E93* in the *Digitor* mid-prepupal mutant is likely to interfere with larval midgut destruction. Moreover, studies have indicated that induction of *E93* is also involved in salivary gland histolysis in response to the ecdysone pulse at 12 hr APF [39,53]. However, at this time point we observed no statistically significant reduction in the levels of *E93* in the *Digitor* mutant (Fig 10E). Nonetheless, an instance of *E93* independent block in salivary gland cell death was reported in *Med24* mutants where a dramatic reduction in *Mdh2* expression was suggested to interfere with salivary gland histolysis [40]. As illustrated in Fig 10G expression of *Mdh2* was also significantly reduced in the *Digitor* mutant pupae at T_14_ providing a possible mechanism for the observed block in salivary gland histolysis. Taken together these results suggest that select subsets of ecdysone-regulated genes as well as *Mdh2* are down regulated during early metamorphosis in the *Digitor* mutant background contributing to defective and incomplete metamorphosis.

### Impaired DNA damage response in the *Digitor* mutant

**Fig 11:**
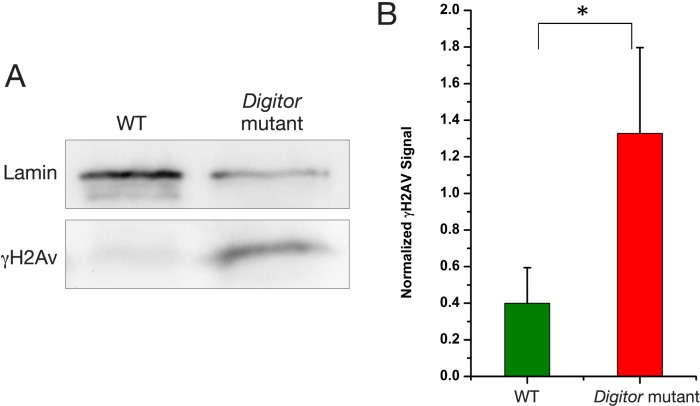
Increased levels of γH2Av in the *Digitor* mutant. (A) Immunoblot comparison of γH2Av levels in protein extracts from wild type (WT) and *Digitor* mutant third instar larvae detected with anti-γH2Av antibody. (B) Average pixel density of the anti-γH2Av antibody-labeled bands normalized to the pixel density of anti-Lamin Dm0 antibody-labeled loading controls from three independent determinations with S.D. The difference between levels in wild type and *Digitor* mutant larvae was statistically significant (Student's two-tailed t-test, P < 0.05)

DNA double-strand break (DSB) repair is an important mechanism for maintaining genomic stability (reviewed in [54]). DSBs trigger activation of the DNA damage response pathway which activates a number of protein kinases including the ATM kinase that rapidly phosphorylate the carboxy-terminus of the histone 2A variant (H2Av) (reviewed in [55]). This phosphorylation is evolutionarily conserved [56] and in *Drosophila* the phosphorylated form is denoted γH2Av with the S137 residue being phosphorylated [17]. Thus, a standard assay for DNA damage detection and recognizing DSBs is to probe for elevated levels of γH2Av using anti-γH2Av antibody [17]. Since the ASCIZ family of proteins may have a general role in modulating ATM kinase signaling [57] we compared γH2Av levels in protein extracts from wild-type and *Digitor* mutant third instar larvae. As illustrated by the immunoblots in Fig 11A there are greatly elevated levels of γH2Av in the mutant larva. We quantified this difference by determining the average pixel density of the anti-γH2Av antibody labeled bands normalized to the pixel density of anti-Lamin Dm0 antibody labeled loading controls. The data from three independent determinations indicate about a 3-fold increase in the levels of γH2Av and concomitant DSBs in the *Digitor* mutant as compared to wild-type (Fig 11B). These data strongly suggest that Digitor/dASCIZ is involved in the DNA damage response in *Drosophila*.

### Increased susceptibility to paraquat induced stress in *Digitor* heterozygous mutants

**Fig 12:**
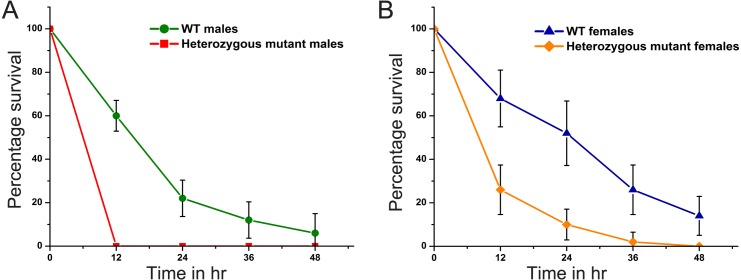
Decreased resistance to paraquat-induced oxidative stress in *Digitor* mutant flies. (A) Survival rates of wild-type and *EP(3)3709* heterozygous male flies 12, 24, 36, and 48 hr after exposure to 20 mM paraquat. (B) Survival rates of wild-type and *EP(3)3709* heterozygous female flies 12, 24, 36, and 48 hr after exposure to 20 mM paraquat. Each data point represents the average survival rate from five independent determinations with S.D.

Since accumulation of DSBs as indicated by elevated levels of γH2Av was 3-fold higher in the *Digitor* homozygous mutants compared to the wild-type controls (Fig 11), we hypothesized that reduced levels of *Digitor/dASCIZ* may also decrease the resistance to paraquat-induced oxidative stress. Paraquat is known to induce DNA damage by generation of free radicals [32,58]. To test this we generated *w*^*1118*^*; EP(3)3709/TM6B*, *Tb*^*1*^ heterozygous mutant flies and compared them to wild-type controls. In the experiments we administered 20 mM paraquat in a 5% sucrose solution to equally aged female and male adult flies from wild-type and *Digitor* heterozygous mutant backgrounds and determined survival in 12 hr intervals for 48 hr. In five independent replicates, a total of 10 flies of each sex and genotype were examined and survival expressed as the percentage of living flies at each time point relative to the total number of starting flies in the vial. As illustrated in Fig 12 the survival rate at each time point was greatly reduced for both male (Fig 12A) and female (Fig 12B) heterozygous mutant flies with males being more affected with no surviving flies after only 12 hr (Fig 12A). These data suggest that Digitor/dASCIZ plays a critical role in conferring resistance to paraquat induced oxidative stress in *Drosophila*.

## Discussion

In this study we present evidence that in *Drosophila* the spindle matrix protein Skeletor interacts with Digitor/dASCIZ, the human ASCIZ ortholog. We detected this interaction in a yeast two-hybrid screen and confirmed it by pull-down assays. The transgenic expression of a mCitrine-labeled Digitor construct shows that Digitor/dASCIZ protein is localized to interband and developmental puffed chromosomal regions during interphase but that it redistributes during mitosis to the spindle region. We have identified and characterized by RT-PCR analysis a P element insertion allele of Digitor/dASCIZ that appears to be a true null allele. When homozygous this allele results in a complete pupal lethal phenotype, indicating that Digitor/dASCIZ is an essential gene. This pupal lethality was partially rescued (31% viability) by the transgenic expression of 3xHA-Digitor-mCitrine in a homozygous Digitor/dASCIZ mutant background. Rescue was not complete, likely due to differences in the levels of expression of the transgene compared with wild type gene expression. Our analysis of the phenotypic consequences of the absence of Digitor/dASCIZ during development combined with its dynamic nuclear localization suggest that Digitor/dASCIZ has multiple roles in *Drosophila* development.

*Digitor/dASCIZ regulation of mitosis*. Previously, Zaytseva et al. [6] using RNAi knockdown of *Digitor/dASCIZ* in the posterior compartment of imaginal wing discs provided evidence that decreased Digitor/dASCIZ leads to impaired mitosis with severe spindle and chromosome alignment defects as well as to reduced wing size. These effects were mainly attributed to a decrease in Dynein light chain (*Cut up*) expression when levels of Digitor/dASCIZ are downregulated [6]. However, we show that Digitor/dASCIZ, in addition to binding to Cut up itself ([6]; this study), has direct physical binding interactions with the spindle matrix protein Skeletor. It has been demonstrated that the *Drosophila* spindle matrix protein Megator homolog Tpr in mammalian cells associates with the Dynein complex during mitosis [59]. Furthermore, live imaging analysis indicated that Digitor/dASCIZ is confined to the spindle region during mitosis at a time after NEB when where there are no diffusion barriers. Thus, these findings suggest the possibility that Digitor/dASCIZ may be playing a direct role in mitotic progression as a member of the spindle matrix and/or Dynein complex in addition to serving as a transcriptional regulator of Dynein light chain expression.

*Digitor/dASCIZ is required for tissue maturation and metamorphosis*. The enrichment and localization of Digitor/dASCIZ to developmental puff regions suggest a role in organ maturation and metamorphosis. Furthermore, the dynamic relocalization of Digitor/dASCIZ from developmental puff regions during heat-shock conditions is compatible with the hypothesis that Digitor/dASCIZ may modulate gene expression at these sites and that this modulation changes during the stress response. An early phenotype we observed in *Digitor* mutant crawling third instar larvae was a severely reduced brain size although overall larval development at this stage appeared normal with the size and weight of the mutant larvae indistinguishable from that of wild-type larvae. While we detected only a small increase in cell death and apoptosis in *Digitor* mutant brains there was a marked decrease in the number of actively dividing cells in the proliferating brain zones suggesting impaired cell proliferation was the main cause of the mutant small brain phenotype. This is in contrast to the findings of Zaytseva et al. [6] indicating that the underlying reason for reduced wing size after Digitor/dASCIZ RNAi knock down was anaphase arrest of mitotic cells caused by Dynein complex-dependent spindle defects leading to enhanced apoptosis. However, considering the pleiotropic effects of *Digitor/dASCIZ* the consequences of its absence is likely to be tissue and context dependent. For example, we provide evidence that the actual programmed cell death of larval salivary gland cells during pupation does not occur in the absence of *Digitor/dASCIZ*. In the *Digitor null* mutant pupation is initiated; however, no eclosion occurs and pupal development is impaired and arrested at various stages. RT-PCR analysis of transcript levels of ecdysone-regulated genes controlling metamorphosis and subsequent eclosion in the ecdysone-signaling cascade showed a significant decrease in some but not all of the genes examined. We therefore propose that the pupal phenotypes in the *Digitor* mutant is a result of changes in expression of a subset of ecdysone-regulated genes as well as *Mdh2* and that *Digitor/dASCIZ* plays a role in metamorphosis by acting as a transcriptional regulator of these genes. However, it should be noted that the experiments do not rule out that these effects are an indirect consequence of timing defects and/or Cut up dysregulation. In mammals ASCIZ also has several ATM-kinase independent developmental functions and is required for B cell maturation as well as for lung, kidney, and brain organogenesis [60–62]. Some of these defects are a consequence of dynein light chain down regulation [8,35]; however, in other cases the effects have been suggested to be caused by modulation of developmental signaling cascades such as the Wnt pathway [62].

*Digitor/dASCIZ is involved in the DNA damage response*. Mammalian ASCIZ has a role in orchestrating ATM-kinase but not NBS1 dependent DNA damage repair in response to agents that perturb chromatin structure such as chloroquine or by osmotic and oxidative stress [35,63]. Here we provide direct evidence for an evolutionarily conserved role for Digitor/dASCIZ in the DNA damage response in *Drosophila*. In the *Digitor* null mutant larvae levels of γH2Av were greatly elevated indicating accumulation of DSBs in the absence of Digitor/dASCIZ. Furthermore, reduced levels of Digitor/dASCIZ decreased the resistance to paraquat-induced oxidative stress resulting in increased mortality in a stress test paradigm. Moreover, Zaytseva et al. [6] have shown that GFP-tagged Digitor/dASCIZ expressed in HeLa cells accumulate within 5 min along tracks of laser-induced oxidative DNA damage reminiscent of the association of human ASCIZ with oxidative DNA damage-induced foci [34].

Thus, in summary we show that Digitor/dASCIZ has multiple developmental functions in *Drosophila* and plays critical roles in regulation of metamorphosis and organogenesis as well as in the DNA damage response. In addition, we provide evidence that Digitor/dASCIZ may be contributing to mitotic progression not just as a regulator of Dynein light chain transcription but also through direct physical interactions with the spindle matrix (e.g. Skeletor). It will be of interest in future studies to further elucidate the many functional roles of Digitor/dASCIZ and their interplay in the highly amenable *Drosophila* model system.

## Supporting Information

S1 Movie Confocal timelapse imaging of transgenically expressed Digitor-mCitrine and tubulin-mCherry in a syncytial *Drosophila* embryo. Confocal timelapse imaging of transgenically expressed Digitor-mCitrine and tubulin-mCherry in a syncytial *Drosophila* embryo (Leica TCS SP5 tandem scanning microscope). The image sequence illustrates the dynamic relationship of Digitor/dASICZ (in green) relative to microtubule spindle formation (in red) through two mitotic cycles. The results suggest that Digitor-mCitrine may associate with the spindle matrix after invasion of microtubules into the nuclear space. The timelapse covers a period of 20 min.
(MOV)

S1 Table Primers for the genes analyzed by RT-PCR. (DOC)

S2 Table Rescue of homozygous *EP(3)3709* lethality by the *3xHA-Digitor-mCitrine* transgene driven by the *Act5C-Gal4* driver. In these crosses the *TM6* chromosome was identified by the *Stubble* marker. Consequently, the experimental genotypes could be distinguished from balanced heterozygotic flies by absence of the *Stubble* marker. The expected Mendelian ratio of non-*Stubble* to *Stubble* flies was 1:2 since *TM6/TM6* is embryonic lethal. The percentage of expected genotypic ratios were calculated as: observed non-*Stubble* flies X 300/total observed flies.
(DOCX)
