# Supplementary material for: Digitor/dASCIZ Has Multiple Roles in Drosophila Development
Source: PLoS One. 2016 Nov 18;11(11):e0166829. doi: 10.1371/journal.pone.0166829 (PMC5115829; doi:10.1371/journal.pone.0166829)
Supplement: S1 Table — (DOC) [file pone.0166829.s002.doc]

**S1 Table. Primers for the genes analyzed by RT-PCR**

**EcR**  Forward 5’-GGAGAACCAATGTGCGATGA- 3′

**EcR**  Reverse 5’-AAGGAAGGTATATTGCGCGC - 3′

**BR-C**  Forward 5’-ACCCAATCGCTCCCACATCCG - 3′

**BR-C**  Reverse 5’-GTTGCTGCCACTGCCAACG - 3′

***E93*** Forward 5’-AGAACGCGTTGCTGAAGAAT-3’

***E93*** Reverse 5’-GATTGCTCTGGCTGATCTCC-3’

***βFTZF1*** Forward 5’-TGGCGTACTTTTAGCGTCCT -3’

***βFTZF1*** Reverse 5’-AATACAAGAATCGATCTTCAAGTGG -3’

**74EF**  Forward 5′-AACGTGCAATCTCTTGAAAGG - 3′

**74EF**  Reverse 5′-AGATAAAGGTACTTAGAGAACGCAAC - 3′

***MDH2*** Forward 5’-CATATCGACACCAAGAGCAAGACCG- 3’

***MDH2*** Reverse 5’-GTGGAGACTCCGAACAGACGCT - 3’

***MED24*** Forward 5’-ATCCTCCAACTCATCTATGTGGCGTGGC-3’

***MED24*** Reverse 5’-CGCACAGTGAATGCTTCAGGTAGTTA-3’

***CG32280*** Forward 5’-ATGAGTAAACCTGGCAGTGCTCC -3’

***CG32280*** Reverse 5’-CTATCGACGGTATCGCCCCA -3’

***Digitor/dASCIZ*** Forward 5’-ATGCACAGCGAAAAACACACG-3’

***Digitor/dASCIZ*** Reverse 5’-CTTGTAGTTCTTGTCCAGGTGGCTC-3’

***Cut-up*** Forward 5’-ATCGAATTCATGTCTGATCGCAAGGCCG-3’

***Cut-up*** Reverse 5'-ATCTCTAGATTAACCGCTCTTAAACAGTAAAATAGCC-3’

***RP-49*** Forward 5’-ATGACCATCCGCCCAGCATAC-3’

***RP-49*** Reverse 5’-GAATCCGGTGGGCAGCATG-3’

***Skeletor*** Forward 5’-GCTCCGATCAAGAAGGTTCCG-3’

***Skeletor*** Reverse 5’-ACTGTAGGACGTTTCACCAGC-3’
