# Supplementary material for: Digitor/dASCIZ Has Multiple Roles in Drosophila Development
Source: PLoS One. 2016 Nov 18;11(11):e0166829. doi: 10.1371/journal.pone.0166829 (PMC5115829; doi:10.1371/journal.pone.0166829)
Supplement: S2 Table — In these crosses the TM6 chromosome was identified by the Stubble marker. Consequently, the experimental genotypes could be distinguished from balanced heterozygotic flies by absence of the Stubble marker. The expected Mendelian ratio of non-Stubble to Stubble flies was 1:2 since TM6/TM6 is embryonic lethal. The percentage of expected genotypic ratios were calculated as: observed non-Stubble flies X 300/total observed flies. (DOCX) [file pone.0166829.s003.docx]

**S2 Table**

**Rescue of homozygous *EP(3)3709*  lethality by the *3xHA-Digitor-mCitrine* transgene driven by the *Act5C-Gal4* driver**

**________________________________________________________________________________________________________________________**

Cross Genotypes (no. of adult flies) % of expected ratio^a^

________________________________________________________________________________________________________________________

*Act5C-Gal4 w^+^/CyO,y^+^; EP(3)3709 w^+^/TM6* X *Act5C-Gal4 w^+^/3xHA-Digitor-mCitrine w^+^; Act5C-Gal4w+/3XHADigitor-mCitrine w+;*

*3xHA-Digitor-mCitrine w^+^/CyO,y^+^; EP(3)3709 w^+^/TM6 EP(3)3709 w^+^/EP(3)3709 w^+^*

*EP(3)3709 w^+^/TM6*

*223 26 31.3%*

________________________________________________________________________________________________________________________

^a^ In these crosses the *TM6* chromosome was identified by the *Stubble* marker. Consequently, the experimental genotypes could be distinguished from balanced heterozygotic flies by absence of the *Stubble* marker. The expected Mendelian ratio of non-*Stubble* to *Stubble* flies was 1:2 since *TM6/TM6* is embryonic lethal. The percentage of expected genotypic ratios were calculated as: observed non-*Stubble* flies X 300/total observed flies.
